# Curcumin reduces expression of Bcl-2, leading to apoptosis in daunorubicin-insensitive CD34^+ ^acute myeloid leukemia cell lines and primary sorted CD34^+ ^acute myeloid leukemia cells

**DOI:** 10.1186/1479-5876-9-71

**Published:** 2011-05-19

**Authors:** Jia Rao, Duo-Rong Xu, Fei-Meng Zheng, Zi-Jie Long, Sheng-Shan Huang, Xing Wu, Wei-Hua Zhou, Ren-Wei Huang, Quentin Liu

**Affiliations:** 1 Department of Hematology, Third Affiliated Hospital, Sun Yat-sen University, 600 Tianhe Road, Guangzhou 510630, P.R. China; 2 Sun Yat-sen Institute of Hematology, 600 Tianhe Road, Guangzhou 510630, P.R. China; 3 Department of Hematology, First Affiliated Hospital, Sun Yat-sen University, 58 Zhongshan II Road, Guangzhou 510080, P.R. China; 4 State Key Laboratory of Oncology in South China, Cancer Center, Sun Yat-sen University, 651 Dongfeng Road East, Guangzhou 510060, P.R. China; 5 School of Life Sciences, Sun Yat-Sen University, No. 135 Xingang Xi Road, Guangzhou 510275, P.R. China

## Abstract

**Background:**

Acute myeloid leukemia (AML) is an immunophenotypically heterogenous malignant disease, in which CD34 positivity is associated with poor prognosis. CD34^+ ^AML cells are 10-15-fold more resistant to daunorubicin (DNR) than CD34^- ^AML cells. Curcumin is a major component of turmeric that has shown cytotoxic activity in multiple cancers; however, its anti-cancer activity has not been well studied in DNR-insensitive CD34^+ ^AML cells. The aim of this study was to therefore to explore curcumin-induced cytotoxicity in DNR-insensitive CD34^+ ^AML cell lines (KG1a, Kasumi-1), DNR-sensitive U937 AML cells, and primary CD34^+ ^AML bone-marrow-derived cells.

**Methods:**

Primary human CD34^+ ^cells were isolated from peripheral blood mononuclear cells or bone marrow mononuclear cells using a CD34 MicroBead kit. The growth inhibitory effects of curcumin were evaluated by MTT and colony-formation assays. Cell cycle distribution was examined by propidium iodide (PI) assay. Apoptosis was analyzed by Wright-Giemsa, Hoechst 33342 and Annexin-V/PI staining assays. The change in mitochondrial membrane potential (MMP) was examined by JC-1 staining and flow cytometry. Expression of apoptosis-related proteins was determined by reverse transcription-polymerase chain reaction and Western blotting. Short interfering RNA (siRNA) against *Bcl-2 *was used in CD34^+ ^KG1a and Kasumi-1 cells incubated with/without DNR.

**Results:**

Curcumin inhibited proliferation and induced apoptosis and G1/S arrest in both DNR-insensitive KG1a, Kasumi-1 and DNR-sensitive U937 cells. Curcumin-induced apoptosis was associated with reduced expression of both Bcl-2 mRNA and protein, subsequent loss of MMP, and activation of caspase-3 followed by PARP degradation. Curcumin synergistically enhanced the cytotoxic effect of DNR in DNR-insensitive KG1a and Kasumi-1 cells, consistent with decreased Bcl-2 expression. Accordingly, siRNA against *Bcl-2 *increased the susceptibility of KG1a and Kasumi-1 cells to DNR-induced apoptosis. More importantly, curcumin suppressed Bcl-2 expression, selectively inhibited proliferation and synergistically enhanced the cytotoxicity of DNR in primary CD34^+ ^AML cells, while showing limited lethality in normal CD34^+ ^hematopoietic progenitors.

**Conclusion:**

Curcumin down-regulates Bcl-2 and induces apoptosis in DNR-insensitive CD34^+ ^AML cell lines and primary CD34^+ ^AML cells.

## Background

Acute myeloid leukemia (AML) is an immunophenotypically heterogenous malignant disease, in which CD34 positivity has been significantly correlated with a lower complete response (CR) rate, drug resistance and poor outcome [1-3]. Treatment of AML has generally consisted of a combination of cytarabine and an anthracycline such as daunorubicin (DNR), or the anthracenedione mitoxantrone [4]. Although conventional chemotherapy regimens induce CR in 65-80% of newly diagnosed AML patients, most patients who achieve a CR relapse within 2 years from diagnosis [5]. At relapse, blast cells usually display a more immature phenotype, with one of the most common antigenic changes being a gain in expression of the stem cell antigen CD34 [6,7]. This is reflected in the resistance of these immature phenotype CD34^+ ^AML progenitors to current chemotherapies.

CD34^+ ^AML cells are 10-15-fold more resistant to DNR than CD34^- ^AML cells [8]. CD34^+ ^KG1a and TF-1 AML cell lines are 30-40 fold more resistant to mitoxantrone than more mature HL-60 and U937 cells, and this resistance appears to be associated with the lack of apoptosis [9]. Increasing evidence indicates that CD34^+ ^AML cells are less sensitive to spontaneous apoptosis and have higher levels of Bcl-2 and Bcl-xl gene and protein expression than the CD34^- ^subpopulation [6,10-12]. CD34 positivity has been reported to be another indicator of poor prognosis in AML [3,12], and use of more effective drugs to eliminate this early immature CD34^+ ^AML cell subpopulation might therefore improve the outcome of AML.

DNR is one of the most commonly used anti-leukemia agents. Bcl-2 overexpression can block DNR-induced apoptosis in more mature U937 AML cells [13]. The anti-apoptotic proteins Bcl-2 and Bcl-xl also contribute to the survival and chemoresistance of quiescent leukemia CD34^+ ^cells [14]. These findings suggest that Bcl-2 plays a critical role in CD34^+ ^AML cell survival and that agents aimed at down-regulating Bcl-2 protein might be effective for the treatment of DNR-insensitive CD34^+ ^AML.

Curcumin, a major yellow pigment in turmeric, has been proven to be a powerful therapeutic drug [15,16]. Curcumin induces apoptosis in a variety of tumor cells, including more mature HL-60 and U937 cell lines, through activation of caspase-3, cytochrome c release, and down-regulation of Bcl-2 [17-20]. Curcumin inhibits proliferation in a variety of cancer cells through targeting multiple cellular signaling pathways [21], including the mitogen-activated protein kinase [22], nuclear factor kappaB [23], phosphoinositide-3 kinase/Akt/mammalian target of rapamycin [24,25], Wnt [26], and Notch-mediated signaling pathways [27]. Curcumin has also been found to be a powerful chemosensitizing agent in tumor cells. It demonstrated no major toxicities in phase I and II clinical studies at doses of up to 8 g/day [28,29]. However, the cytotoxic effects of curcumin in DNR-insensitive CD34^+ ^immature AML cells remain unclear.

In this study, we examined the cytotoxic efficiency and molecular mechanisms underlying the anticancer activity of curcumin in both DNR-insensitive CD34^+ ^immature AML cell lines and in primary CD34^+^AML cells.

## Methods

### Materials

Curcumin (Sigma, St. Louis, MO) was dissolved in dimethyl sulfoxide (DMSO) to prepare a 100-mM stock solution that was stored at -20°C. DNR was purchased from Pharmacia & Upjohn SpA (Milan, Italy). Annexin-V assay kit was purchased from Molecular Probes (Eugene, OR, USA). Anti-cleaved PARP, cleaved caspase-3, and Bcl-2 antibodies were purchased from Cell Signaling Technologies (Beverly, MA, USA). Anti-GAPDH antibody and goat anti-rabbit/mouse-horseradish peroxidase (HRP)-conjugated secondary antibody were purchased from Protein Tech Group (Chicago, IL, USA). JC-1 kit was purchased from Beyotime (China). CD34-PE and IgG1-PE monoclonal antibodies were purchased from BD Biosciences (San Jose, CA, USA). CD34 MicroBead kit was purchased from Miltenyi biotec (Auburn, CA, USA).

### Cell lines, primary samples, and cell culture

KG1a and Kasumi-1 cell lines were obtained from Deutsche Sammlung von Mikroorganismen und Zellkulturen GmbH (DSMZ) (Braunschweig, Germany) and grown in RPMI 1640 medium (Gibco; Invitrogen, Carlsbad, CA, USA) supplemented with 20% (v/v) fetal bovine serum (FBS; Hyclone, Logan, UT). According to immunological studies by DSMZ and others [30,31], KG1a and Kasumi-1 cells are characterized by high expression of CD34 surface antigen. U937 cells were obtained from the American Type Culture Collection (ATCC) and grown in RPMI 1640 medium supplemented with 10% FBS. Cells were cultured at 37°C in a humidified atmosphere containing 5% CO_2_. Control cultures received an equivalent amount of DMSO only. Bone marrow mononuclear cells (BMMCs) or mobilized peripheral blood mononuclear cells (PBMCs) were obtained from 9 newly diagnosed AML patients and 8 healthy donors. All donors provided written informed consent, and the study had the approval of the Institute Research Ethics Committee at Sun Yan-sen University, in accordance with the Declaration of Helsinki. Patient characteristics are shown in Table 1. PBMCs and BMMCs were enriched by Ficoll-Hypaque density gradient centrifugation and isolated using a CD34 MicroBead kit. BMMCs and PBMCs were stained with PE-conjugated anti-CD34 to determine the purity of CD34^+ ^cells.

**Table 1:** Characteristic of patients

Patient#	Age/Sex	FAB	WBC(*109/L)	%CD34 in BMC*	Source	Cytogenetics
P1	35 Y/M	M2	9.09	67.3	BM	46 XY
P2	60 Y/M	M2b	15.80	72.1	BM	46 XY, t(8;21),AML1/ETO #
P3	46 Y/M	M5	12.00	56.0	BM	46 XY
P4	17 Y/M	M5b	3.39	89.1	BM	46 XY
P5	28 Y/M	M2b	11.37	76.3	BM	46 XY, t(8;21),AML1/ETO #
P6	20 Y/M	M1	10.03	70.1	BM	46 XY
P7	78 Y/M	M4	101.08	52.1	PB	46 XY inv (16)(p13q22)
P8	72 Y/F	M2a	1.95	31.5	PB	46 XX
P9	54 Y/M	M1	103.79	62.8	BM	46 XY

*P *patient, *Y *year, *M *male, *FAB *French-American-British, *WBC *white blood cells, *BMC *bone marrow cells, *BM *bone marrow, *PM *peripheral blood.*Percentage of CD34^+ ^cells in bone marrow cells of AML patients before sorting. ^# ^The t (8; 21) (q22; q22) chromosomal translocation gives rise to the AML1/ETO fusion oncoprotein

### MTT assay

Viability was assessed by MTT assay. Briefly, 1.0×10^4 ^cells were incubated in triplicate in a 96-well plate in the presence or absence of the indicated test samples in a final volume of 0.2 ml for various lengths of time at 37°C. Thereafter, 20 μl MTT solution (5 mg/ml in PBS) was then added to each well. After 4-h incubation at 37°C, 150 μl DMSO was added. Finally the plates were shaken and the optical density at 490 nm was measured using a multiwell plate reader (Microplate Reader; Bio-Rad, Hercules, CA). Percent cell viability was calculated as cell viability of the experimental samples/cell viability of the control samples × 100. At least three independent experiments were performed.

### Colony-forming assay

Treated and untreated cells were cultured in RPMI 1640 medium supplemented with 0.9% methylcellulose and 20% FBS at 37°C in 5% CO_2_. The colonies (containing 50 or more cells) were counted by light microscopy after 14 days. All semi-solid cultures were performed in triplicate. Three independent experiments were performed.

### Wright-Giemsa staining

Morphological signs of apoptosis were detected by Wright-Giemsa staining. Cells were treated with 0-80 μM curcumin for 24 h. Smears of control and treated cells were stained with Wright-Giemsa solution for 25 min, rinsed with distilled water and air dried. Cell morphology was studied by light microscopy.

### Hoechst 33342 staining

Nuclear fragmentation was examined by Hoechst 33342 (Sigma). Cells treated with 0-80 μM curcumin for 24 h were washed and stained with Hoechst 33342 (10 μg/ml) for 15 min at 37°C. Slides were viewed using a fluorescence microscope.

### Measurement of apoptosis by Annexin V analysis

An Annexin V-binding assay was used according to the manufacturer's instructions. Briefly, approximately 5×10^5^/ml cells in 6-well plates were treated with various concentrations of the indicated test samples. The cells were harvested and used for Annexin V-Alexa Fluor-488/PI staining. The stained cells were analyzed by flow cytometry to determine the percentages of AnnexinV^+^/PI^- ^(early apoptosis) and AnnexinV^+^/PI^+ ^(late apoptosis) cells.

### Cell cycle analysis

Cell cycle was analyzed by flow cytometry. Approximately 5 × 10^5^/ml cells in 6-well plates were treated with various concentrations of curcumin for 24 h. Cell cycle analysis was performed using the CycleTEST™ PLUS DNA kit (BD Biosciences).

### Detection of mitochondrial membrane potential (MMP, Δψm) using JC-1

MMP was estimated by flow cytometry after staining with JC-1 fluorescent dye. When the cell is in a normal state, MMP is high and JC-1 predominantly appears as red fluorescence. When the cell is in an apoptotic or necrotic state, the MMP is reduced and JC-1 appears as a monomer indicated by green fluorescence. A change in the florescence from red to green indicates a decrease in the MMP. Approximately 5×10^5^/ml cells in 6-well plates were treated with various concentrations of curcumin for 24 h. The cells were then washed with PBS and incubated with JC-1 working solution for 20 min at 37°C in the dark. Cells were washed with PBS and resuspended in 500 μl PBS. The stained cells were analyzed by flow cytometry to determine the change in the florescence from red to green.

### RNA isolation and semiquantitative reverse transcription-polymerase chain reaction (RT-PCR)

Total RNA was extracted using Trizol isolation reagent (Invitrogen, USA). Reverse transcription was performed using a reverse transcriptase first strand cDNA synthesis kit (Takara, Japan). The sequences of the sense and antisense primers were: 5'-CTGGTGGACAACATCGC-3' (sense) and 5'-GGAGAAATCAAACAGAGGC-3' (anti-sense) for Bcl-2, 5'-TGACTTTTCCTGTGAACTCT-3' (sense) and 5'-GCCTTTCATTCGTATCAAGA-3' (anti-sense) for c-IAP-1, 5'-GCAGGGTTTCT TTATACTG-3' (sense) and 5'-TGTCCCTTCTGTTCTAACAG-3' (anti-sense) for XIAP [32], 5'-GTGGACATCCGCAAAGAC-3' (sense) and 5'-GAAAGGGTGTAA CGCAACT-3' (anti-sense) for β-actin. The PCR conditions were as follows: for c-IAP-1 and XIAP, 94°C for 1 min, 62°C for 1 min, and 72°C for 1 min; for Bcl-2, 94°C for 30 s, 62°C for 30 s, 72°C for 10 s; and for β-actin: 94°C for 30 s, 55°C for 30 s, 72°C for 1 min. Thirty cycles of amplification were used. PCR (10 μl) products were analyzed by electrophoresis on 2% (w/v) agarose gel.

### Western blot analysis

Total cellular proteins were isolated with lysis buffer (20 mM Tris, pH 7.5; 150 mM NaCl; 0.25% NP40; 2.5 mM sodium pyprophosphate; 1 mM EGTA, 1 mM EDTA; 1 mM β-glycerophosphate; 1 mM Na_3_VO_4_; 1 mM PMSF; 1 μg/ml leupeptin). Equal amounts of protein were subjected to 10% or 15% sodium dodecyl sulfate-polyacrylamide gel electrophoresis and transferred to nitrocellulose membranes. The membranes were treated with primary antibodies overnight at 4°C and incubated with a HRP-conjugated anti-mouse or anti-rabbit secondary antibody at room temperature for 1 h. The protein bands were visualized using an enhanced chemiluminescence reagent (Pierce Biotechnology, USA), according to the manufacturer's instructions.

### Short interfering RNA (siRNA) transfection

KG1a and Kasumi-1 cells were seeded onto 6-well plates for 24 h before transfection. Control scrambled siRNA was synthesized and purchased from GenePharma (Shanghai Co. Ltd., China). SiRNA Bcl-2 (50 nM): 5'-GGGAGAUAGUGAUGAAG UAUU-3' [33] or control scramble sequences were transfected using Lipofectamine 2000 reagent (Invitrogen), according to the manufacturer's protocol. Briefly, for each well, 5 μl Lipofectamine 2000 was diluted in 250 μl Opti-MEM medium (Invitrogen). The mixture was gently added to a solution containing siRNA in 250 μl Opti-MEMI medium and incubated for 20 min. The mixture was then added to the plates. After transfection with siRNA for 24 h, cells were harvested for further assay.

### Statistical analysis

Data were presented as mean ± SD. One-way ANOVA followed by Bonferroni multiple comparison was performed to assess the differences between two groups under multiple conditions. If the data failed the normality test, the Kruskal-Wallis one-way ANOVA on ranks was used. A value of p < 0.05 was considered statistically significant. Both Calcusyn software (Biosoft, Ferguson, MO, USA) [34,35] and Jin's formula [36] were used to evaluate the synergistic effects of drug combinations. Jin's formula is given as: Q = Ea + b/(Ea + Eb-Ea × Eb). Ea+b represents the cell proliferation inhibition rate of the combined drugs, while Ea and Eb represent the rates for each drug respectively. A value of Q = 0.85-1.15 indicates a simple additive effect, while Q > 1.15 indicates synergism. Combination index (CI) plots were generated using CalcuSyn software. A value of CI < 1 indicates synergism.

## Results

### CD34^+ ^KG1a and Kasumi-1cells were insensitive to DNR

**Figure 1:**
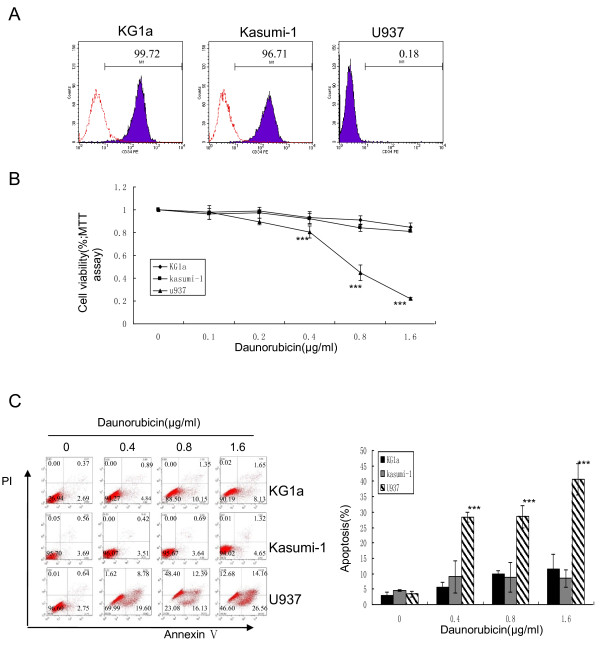
**CD34^+ ^KG1a and Kasumi-1cells were insensitive to DNR**. (**A**) KG1a, Kasumi-1 and U937 cells were stained with PE-conjugated CD34 antibody and subjected to flow cytometry to determine the purity of CD34^+ ^cells. (**B, C**) These three cell lines were treated with different concentrations of DNR for 48 h. MTT assay (**B**) was performed as described in "Methods" and apoptosis (**C**) was assessed by Annexin V/PI assays. Cells in the lower right quadrant represent early apoptosis and those in the upper right quadrant represent late apoptosis. The graph displays the means ± SD of three independent experiments. * p < 0.05, ** p < 0.01, *** p < 0.001 (compared with control).

KG1a, Kasumi-1 and U937 AML cells were stained with PE-conjugated CD34 antibody and subjected to flow cytometry to determine the purity of CD34^+ ^cells. The percentages of CD34^+ ^cells were 99.43 ± 0.60% in KG1a cells, 96.67 ± 0.11% in Kasumi-1 cells, but CD34^+ ^was absent in U937 cells (Figure 1A). After treatment of these three cell lines with different concentrations of DNR for 48 h, MTT and apoptosis analyses showed that DNR inhibited proliferation and induced apoptosis in more mature U937 cells, but not in immature CD34^+ ^KG1a or Kasumi-1 cell lines (Figure 1B, C). This was in accord with previous studies indicating that CD34^+ ^AML cells were insensitive to DNR. The concentration of DNR used in this study was clinically achievable in patients [37,38].

### Curcumin suppressed cell growth and induced G1/S cell cycle arrest in both DNR-insensitive and -sensitive AML cell lines

**Figure 2:**
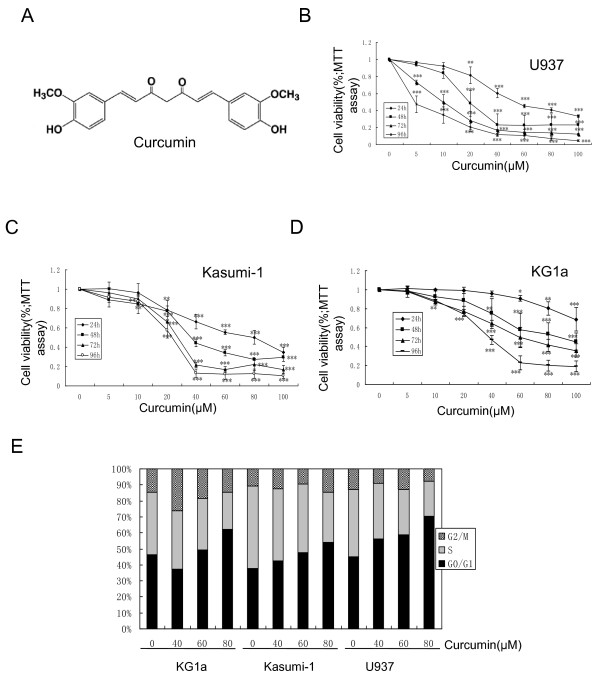
**Curcumin suppressed cell growth and induced G1/S arrest**. (**A**) Structure of curcumin. (**B, C, D**) KG1a, Kasumi-1 and U937 cell lines were treated with different concentrations of curcumin for 24, 48, 72, and 96 h. MTT assay was performed. (**E**) These three cell lines were treated with different concentrations of curcumin for 24 h and analyzed for DNA content by flow cytometry, as described in "Methods." The bar represents means ± SD of three independent experiments.

KG1a, Kasumi-1 and U937 cell lines were exposed to curcumin (0-100 μM) for 24, 48, 72 and 96 h. The cytotoxic effects of curcumin were determined by MTT assay. Curcumin had a significant cytotoxic effect in all tested cell lines in both dose- and time-dependent manners (Figure 2B, C, D). The IC_50 _values at 24, 48, 72, and 96 h were 230.5, 86.9, 60.0, and 35.7 μM for KG1a, 68.5, 46.6, 28.8, and 23.5 μM for Kasumi-1, and 58.3, 26.0, 10.6, and 4.4 μM for U937 cells, respectively. The antiproliferative effects of curcumin in these cell lines were further determined using clonogenic assays. Curcumin inhibited clonogenic growth in a dose-dependent manner, and completely inhibited colony formation at a dose as low as 20 μM (Figure S1A, B, Additional file 1).

Cell cycle distributions in KG1a, Kasumi-1, and U937 cells were examined after treatment with curcumin for 24 h. As shown in Figure 2E, treatment of KG1a cells with 80 μM curcumin resulted in a significant increase in the percentage of cells in the G1 phase, from 46-62%, and a decrease in the percentage of cells in the S phase, from 39-23%. Similar results were found for Kasumi-1 and U937 cells. These results demonstrated that curcumin induced G1/S arrest in both DNR-insensitive and -sensitive AML cell lines.

### Curcumin induced apoptosis through activation of caspase-3 followed by PARP degradation in both DNR-insensitive and -sensitive AML cell lines

**Figure 3:**
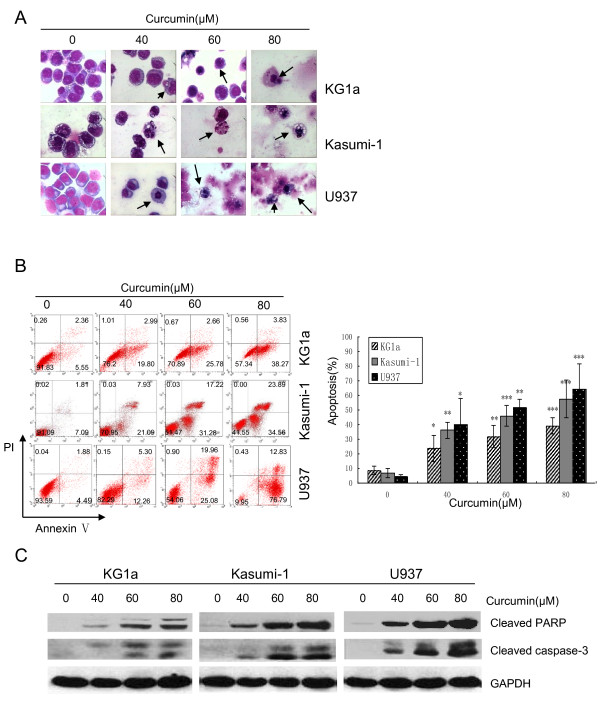
**Curcumin induced apoptosis through activation of caspase-3 followed by PARP degradation**. KG1a, Kasumi-1 and U937 cells were incubated with indicated concentrations of curcumin for 24 h. **(A) **Cells were stained with Wright-Giemsa and then examined under a light microscope. Arrows indicate apoptotic cells (magnification ×400). (**B**) Cells were stained with Annexin V/PI to analyze apoptotic cell populations. The graph displays the means ± SD of four independent experiments. (**C**) Western blotting analysis showed cleaved caspase-3 (17, 19 kDa) and cleaved PARP (89 kDa) fragment. Three independent experiments were performed with similar results, and representative data are shown.

To determine if growth inhibition induced by curcumin was a result of apoptosis, the pro-apoptotic effect was examined using Wright-Giemsa, Hoechst 33342 and Annexin-V/PI staining. Both Wright-Giemsa and Hoechst 33342 staining showed that curcumin induced morphological changes such as cell shrinkage and nuclear condensation, which are typical characteristics of apoptosis (Figure 3A; Figure S2A, Additional file 2). These morphological changes were confirmed by flow cytometry. Treatment with curcumin at 40 μM for 24 h resulted in apoptosis rates of 23.5 ± 8.8%, 36.1 ± 5.3%, and 40.1 ± 17.8% in KG1a, Kasumi-1 and U937 cells, respectively (Figure 3B). Western blotting analysis further showed that curcumin induced caspase-3 activation and PARP cleavage, two hallmarks of apoptosis (Figure 3C). Both Annexin-V/PI and Western blotting showed that curcumin induced apoptosis in a dose-dependent manner. U937cells were the most sensitive to curcumin-induced apoptosis, followed by Kasumi-1, then KG1a cells.

### Curcumin decreased Bcl-2 mRNA and protein levels and reduced MMP in both DNR-insensitive and -sensitive AML cell lines

**Figure 4:**
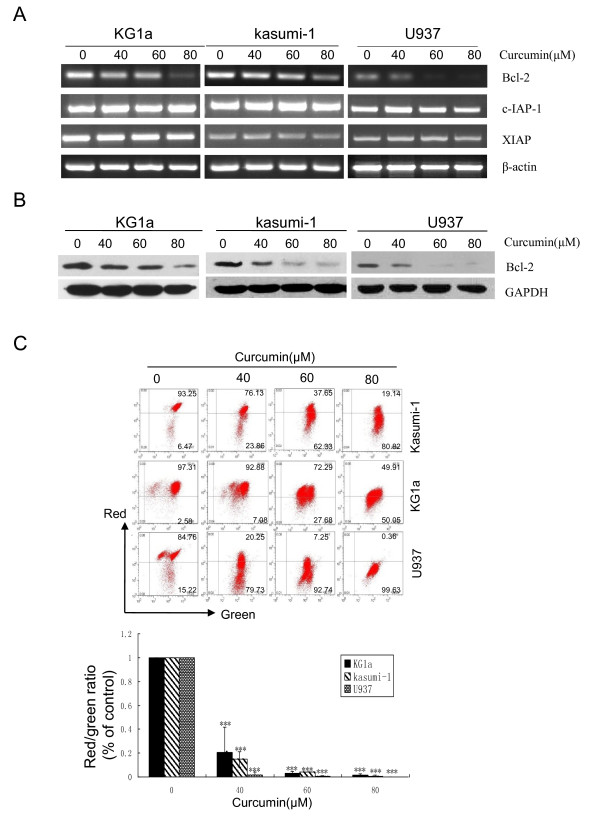
**Curcumin decreased Bcl-2 mRNA and protein levels and caused the loss of MMP**. KG1a, Kasumi-1 and U937 cells were exposed to different concentrations of curcumin for 24 h. (**A**) The effects on Bcl-2, c-IAP-1, and XIAP mRNA levels were determined by RT-PCR. (**B**) The effect on Bcl-2 protein levels was determined by Western blotting assay. Three independent experiments were performed with similar results, and representative data are shown. (**C**) MMP was estimated by flow cytometry showing decrease in the red to green fluorescence ratio. The results shown are representative of three independent experiments. The bar represents mean ± SD of three independent experiments.

The mechanisms underlying curcumin-induced apoptosis were investigated. The IAP and Bcl-2 family play an important role in the regulation of cell apoptosis, and the effects of curcumin on mRNA levels of c-IAP-1, XIAP and Bcl-2 were therefore assessed by RT-PCR. As shown in Figure 4A, Bcl-2 mRNA levels were significantly down-regulated in both DNR-insensitive AML cell lines (KG1a and Kasumi-1) and in DNR-sensitive U937 cells, while the levels of c-IAP-1 and XIAP were unchanged. Western blotting also demonstrated that curcumin significantly down-regulated Bcl-2 protein levels in a dose-dependent manner (Figure 4B). These results suggest that down-regulation of Bcl-2 could contribute to curcumin-induced apoptosis.

Disruption of the function of Bcl-2 protein leads to permeabilization of the mitochondrial membrane [39]. We therefore investigated the effects of curcumin on MMP using JC-1 fluorescent dye and flow cytometry. Exposure of the three cell lines to increasing doses of curcumin for 24 h led to a significant reduction in the MMP (Figure 4C). These results suggest that curcumin-induced apoptosis is mitochondria-dependent.

### Curcumin synergistically enhanced the cytotoxic effect of DNR in DNR-insensitive KG1a and Kasumi-1 cells, associated with down-regulation of Bcl-2

**Figure 5:**
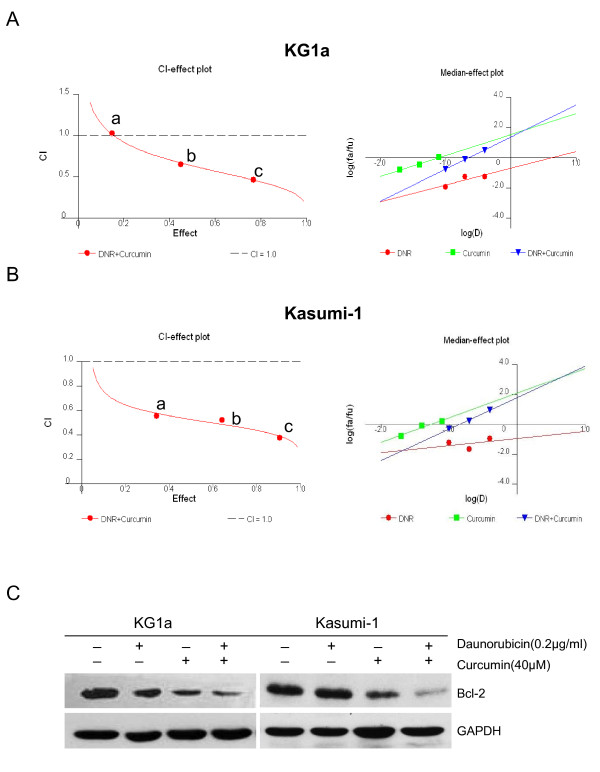
**Curcumin synergistically enhanced the cytotoxic effects of DNR associated with down-regulation of Bcl-2**. KG1a and Kausumi-1cells were exposed to different concentrations of curcumin, DNR, or their combination as indicated, for 48 h. (**A, B**) CI-effect plots and median-effect plots were generated using CalcuSyn software. The points a, b, and c represent CI values for the combinations 20, 40, and 80 μM curcumin with 0.1, 0.2, and 0.4 μg/ml DNR in a constant ratio, respectively. The CI values at ED_50_, ED_75_, ED_90 _were 0.667, 0.490, and 0.364 for KG1a cells and 0.529, 0.456, and 0.394 for Kasumi-1 cells, respectively. (**C**) Bcl-2 protein levels were determined by Western blotting assay. Three independent experiments were performed with similar results, and representative data are shown.

To determine if curcumin could enhance the cytotoxic activity of DNR, DNR-insensitive KG1a and Kasumi-1 cells were cultured with combinations of these two drugs at different doses but in a constant ratio (curcumin to DNR: 20 μM to 0.1 μg/ml, 40 μM to 0.2 μg/ml, and 80 μM to 0.4 μg/ml, respectively) for 48 h, as shown in Figure 5A, B and Table S1 (Additional file 3). Both CalcuSyn software and Jin's formula were used to determine synergy, and the results were consistent. With the exception of co-treatment of KG1a cells with 20 μM curcumin and 0.1 μg/ml DNR, which showed an additive effect (CI = 1.03, Q = 0.99), co-treatment with other doses in KG1a cells and with all doses in Kasumi-1 cells exhibited synergistic effects. For example, the combination of 40 μM curcumin with 0.2 μg/ml DNR in KG1a cells caused growth inhibition of 45.12%, compared to curcumin (26.31%) or DNR (5.47%) alone, indicating synergism (CI = 0.654, Q = 1.49). Notably, co-treatment with 40 μM curcumin and 0.2 μg/ml DNR caused more attenuation of Bcl-2 protein levels than treatment with either agent alone (Figure 5C).

### Suppression of Bcl-2 with siRNA induced apoptosis and increased the susceptibility of KG1a and Kasumi-1 cells to DNR-induced apoptosis

**Figure 6:**
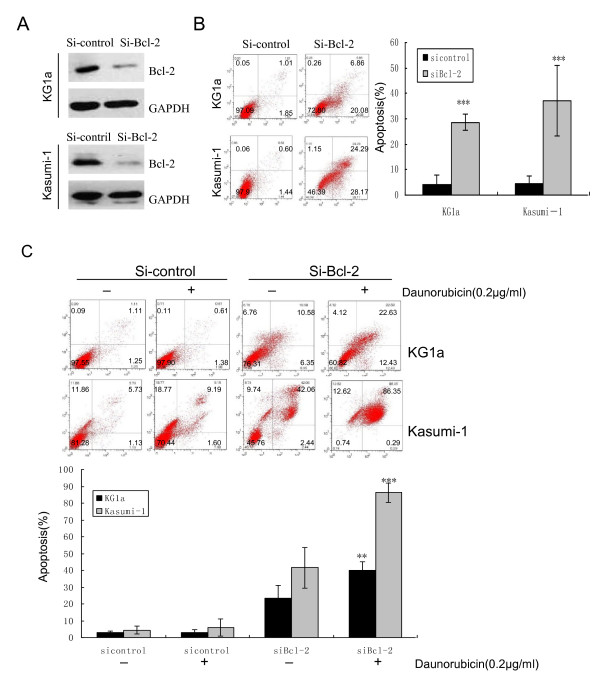
**Suppression of Bcl-2 with siRNA induced apoptosis and increased susceptibility to DNR**. KG1a and Kasumi-1cells were transfected with siRNA Bcl-2 or siRNA control for 24 h. (**A**) Bcl-2 protein levels were determined by Western blotting assay. (**B**) Cells were stained with Annexin V/PI to analyze apoptotic cell populations. The graph displays the means ± SD of three independent experiments. (**C**) KG1a and Kasumi-1 cells were transfected with siRNA Bcl-2 or siRNA control for 24 h, and then treated with DNR (0.2 μg/ml) for 48 h. Cells were stained with Annexin V/PI to analyze apoptotic cell populations. The bar represents mean ± SD of three independent experiments. ** p < 0.01, *** p < 0.001 (compared with control).

To clarify if down-regulation of Bcl-2 by curcumin plays an important role in this synergistic effect, Bcl-2 expression was suppressed by siRNA and the effect on apoptosis and DNR sensitivity was examined by flow cytometry. Bcl-2 siRNA-induced apoptosis in 24 h (28.58% in KG1a cells, 37.12% in Kasumi-1 cells) was similar to that in curcumin-treated KG1a (31.71%, 60 μM, Figure 3B) and Kasumi-1 cells (36.10%, 40 μM, Figure 3B), respectively (Figure 6A, B). As shown in Figure 6C, suppression of Bcl-2 by siRNA increased the susceptibility of these cell lines to DNR-induced apoptosis (40.15% in KG1a cells and 86.23% in Kasumi-1 cells), compared to DNR only (3.17% in KG1a cells, 5.94% in Kasumi-1 cells). These results suggest that suppression of Bcl-2 could contribute to curcumin-induced apoptosis and the synergistic effect of curcumin and DNR.

### Curcumin was effective against primary CD34^+ ^AML cells

**Figure 7:**
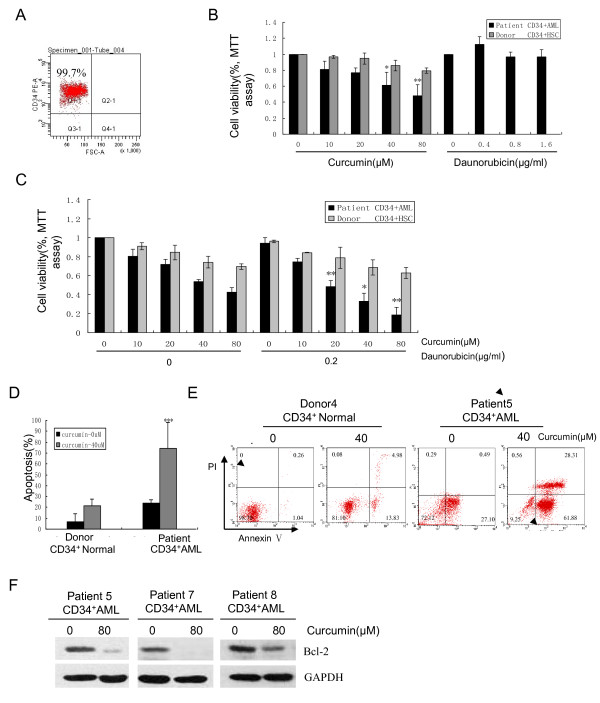
**Curcumin was effective against primary CD34^+ ^AML cells**. (**A**) Primary CD34^+ ^cells isolated from BMMCs or PBMCs of 9 AML patients and 8 healthy donors were isolated and subjected to flow cytometry to determine the purity of CD34^+ ^cells. (**B**) 3 CD34^+ ^AML and 3 CD34^+ ^normal samples were treated with different concentrations of curcumin (0, 10, 20, 40, and 80 μM) for 24 h. The same CD34^+ ^AML samples were also exposed to DNR (0, 0.4, 0.8, and 1.6 μg/ml) for 24 h. MTT assay was performed. The bar represents mean ± SD of three independent experiments. (**C**) Another set of 3 CD34^+ ^AML samples and 3 CD34^+ ^normal samples were exposed to different concentrations of curcumin, DNR, and their combination as indicated, for 48 h. MTT assay was performed. * P < 0.05, ** P < 0.01 (compared with either curcumin or DNR alone). (**D, E**) 4 CD34^+ ^AML samples and 3 CD34^+ ^normal samples were treated with 0 and 40 μM curcumin for 24 h. Apoptosis was assessed by AnnexinV/PI assay. The graph displays the mean ± SD of four independent experiments (**D**). A representative figure is shown (**E**). (**F**) 3 CD34^+ ^AML samples were treated with 0 and 80 μM curcumin for 24 h. Bcl-2 protein levels were determined by Western blotting assay.

The cytotoxic effects of either curcumin and/or DNR on primary CD34^+^AML cells were also examined. CD34^+ ^cells were sorted from BMMCs or PBMCs from 9 AML patients and 8 healthy donors. The sorted samples yielded more than 95% CD34^+ ^cells with greater than 90% viability, determined by trypan blue exclusion (Figure 7A). The antiproliferative effects of curcumin on CD34^+ ^cells from 3 AML patients (patients 1, 2, 3) and 3 healthy donors (donors 1, 2, 3) were determined by MTT assay, and compared with the results of DNR treatment. CD34^+ ^cells were treated with curcumin (0, 10, 20, 40, 80 μM) or DNR (0, 0.4, 0.8, 1.6 μg/ml) for 24 h. Curcumin significantly inhibited proliferation of CD34^+ ^AML cells, but only exhibited modest lethality in normal CD34^+ ^hematopoietic progenitors (Figure 7B). However, CD34^+ ^cells derived from the 3 AML patients were insensitive to DNR (Figure 7B). Synergy between curcumin and DNR was examined in another set of 3 AML patients (patients 7, 8, 9) and 3 healthy donors (donors 6, 7, 8). CD34^+ ^cells were treated with curcumin (0, 10, 20, 40, 80 μM) and/or DNR (0.2 μg/ml) for 48 h. Curcumin at 20, 40, or 80 μM synergistically enhanced the cytotoxic effect of DNR (0.2 μg/ml) in CD34^+ ^AML cells, with Q values of 1.60, 1.35 and 1.33, respectively. Normal CD34^+ ^progenitors were less susceptible to the combined toxic effects (Figure 7C). 4 AML patients (patients 4, 5, 6, 7) and 3 donors (donors 4, 5, 6) yielded sufficient numbers of cells for apoptosis assay by flow cytometry. As shown in Figure 7D, E, curcumin induced significant apoptosis in CD34^+ ^AML cells, but minimal apoptosis in normal CD34^+ ^hematopoietic progenitors. 3 AML samples (patients 5, 7, 8) with sufficient cell numbers were further analyzed for Bcl-2 protein expression by Western blotting assay. A dose of 80 μM curcumin was used in primary CD34^+ ^AML cells, because curcumin significantly down-regulated the Bcl-2 protein levels in CD34^+ ^AML cell lines at 80 μM. The results showed that treatment with 80 μM curcumin significantly down-regulated Bcl-2 protein levels (Figure 7F).

## Discussion

CD34 positivity has been reported to be an indicator of poor prognosis in AML [3]. In the present study, we evaluated the cytotoxicity of curcumin in DNR-insensitive CD34^+ ^AML cell lines (KG1a and Kasumi-1) and in CD34^+ ^primary AML samples. We showed that curcumin selectively induced apoptosis in KG1a and Kasumi-1 cell lines, as well as in primary CD34^+ ^AML cells, in association with down-regulation of Bcl-2 expression. Importantly, co-treatment with curcumin and DNR synergistically inhibited proliferation, consistent with decreased Bcl-2 expression. Accordingly, suppression of Bcl-2 with siRNA increased the susceptibility of KG1a and Kasumi-1 cells to DNR-induced apoptosis. These results provide the first evidence for the ability of curcumin to overcome insensitivity to DNR by down-regulation of Bcl-2 in CD34^+ ^AML progenitors.

Insensitivity to chemotherapy is a major obstacle to cancer treatment. CD34^+ ^cell lines display natural resistance to mitoxantrone associated with an absence of apoptosis [9], giving these immature myeloid leukemia cells a survival advantage over the more mature leukemia hematopoietic compartment. Curcumin induced apoptosis in more mature HL-60 AML cells by releasing cytochrome c and activating caspase-3 [18]. The results of the present study demonstrated that curcumin induced apoptosis in both DNR-sensitive U937 cells and DNR-insensitive KG1a and Kasumi-1 cells via the intrinsic apoptosis pathway involving down-regulation of Bcl-2 protein, loss of MMP and activation of caspase-3, followed by PARP degradation. Furthermore, suppression of Bcl-2 with siRNA caused significant apoptosis, similar to that seen in curcumin-treated cells, suggesting an important role for Bcl-2 in curcumin-induced apoptosis in these CD34^+^AML cell lines.

Accumulating evidence has shown that curcumin potentiates the effects of chemotherapeutic drugs such as bortezomib, cisplatin, and 5-fluorouracil plus oxaliplatin (FOLFOX) *in vitro *and *vivo *[40-42]. Notably, Yu et al. revealed that curcumin, either alone or together with FOLFOX, could effectively eliminate FOLFOX-resistant colon cancer stem cells (CSCs) [42]. CSCs have been proposed to be responsible for disease progression or relapse following conventional therapy [43], and the results of the current study suggest that curcumin could act as a potentially powerful chemosensitizing agent in tumor cells, including CSCs. A recent study indicated that the combination of curcumin with carnosic acid also produced a synergistic antiproliferative effect on KG1a cells; however, this synergism was not associated with alterations in Bcl-2 levels [44]. In contrast, our study demonstrated that curcumin synergistically enhanced the cytotoxic effects of DNR in association with decreased Bcl-2 expression in KG1a and Kasumi-1 cells. Accordingly, siRNA against *Bcl-2 *increased the susceptibility of these CD34^+ ^cell lines to DNR-induced apoptosis, indicating that Bcl-2 down-regulation played an important role in this curcumin-induced synergistic effect.

Anti-apoptotic Bcl-2 contributes to the survival and chemoresistance of quiescent leukemia CD34^+ ^cells [14]. CD34^+ ^AML cells have higher levels of Bcl-2 gene and protein than CD34^- ^AML cells [6]. DNR-induced apoptosis can be blocked by Bcl-2 overexpression in DNR-sensitive CD34^- ^U937 cells [13]. Conversely, suppression of Bcl-2 expression with siRNA enhanced DNR-induced apoptosis in DNR-insensitive CD34^+ ^KG1a and Kasumi-1 cells. These results suggest that high levels of Bcl-2 expression could contribute to DNR-insensitivity, and that down-regulation of Bcl-2 by curcumin could be a molecular mechanism whereby curcumin can overcome the insensitivity of CD34^+ ^AML cells to DNR.

We further demonstrated that primary CD34^+ ^AML cells also underwent proliferation inhibition and apoptosis with curcumin exposure. This effect was replicated in 9 individual patient samples representative of different French-American-British (FAB) classifications. Furthermore, curcumin also suppressed Bcl-2 expression and synergistically enhanced DNR cytotoxicity in primary CD34^+ ^AML cells. These primary cells with different FAB classifications represented a broad cross-section of common AML types, suggesting that down-regulation of Bcl-2 and induction of apoptosis by curcumin could be a common death mechanism in CD34^+ ^AML cells.

Several phase I and phase II clinical trials have indicated the potential therapeutic efficacy and lack of toxic side effects associated with curcumin [28,29]. However, its poor bioavailability has limited its use for the treatment of cancers outside the gastrointestinal tract [45]. Modern techniques such as the use of synthetic analogs, derivatives, different formulations and heat-solubilized curcumin have been explored with the aim of improving its bioavailability [46-48]; e.g., the water solubility of curcumin could be increased 12-fold by heating, without destroying its biological activity [47,48].

## Conclusion

In summary, this study demonstrated a potential new mechanism whereby curcumin could overcome DNR insensitivity by down-regulating Bcl-2 in both CD34^+ ^AML cell lines and in primary CD34^+ ^AML cells. Curcumin, either alone or in combination with DNR, could thus be a potential anti-leukemic agent for the treatment of DNR-insensitive CD34^+ ^AML cells.

## Abbreviations

MAPK: mitogen-activated protein kinase; NF-κB: nuclear factor kappa B; mTOR: mammalian target of rapamycin; PI3K: phosphoinositide 3-kinase; Bcl-2: B cell lymphoma 2; IAP: inhibitor of apoptosis protein.

## Competing interests

The authors declare that they have no competing interests.

## Authors' contributions

JR carried out protein studies, apoptosis analysis, statistical analysis, and drafted the manuscript. FZ and ZL performed the protein studies, apoptosis analysis. SH, XW and WZ participated in the statistical analysis. QL and RH conceived of the study, and participated in its design and coordination. DX contributed effort in designing, performing, and analyzing the results and conclusion. All authors read and approved the final manuscript.

## Supplementary Material

Additional file 1 **Figure S1 Curcumin inhibited clonogenic growth**. (**A**) The colonies (containing ≥50) were counted after 14 days by light microscopy (magnification ×40). (**B**) Results show numbers of colonies in the curcumin-treated group expressed as a percentage of number of colonies in the DMSO-treated group. The graph displays the means ± SD of three independent experiments. Click here for file

Additional file 2 **Figure S2 Morphological changes in nuclei in curcumin-treated cells**. **(A) **KG1a, Kasumi-1 and U937 cells were incubated with the indicated concentrations of 0, 40, 60, and 80 μM curcumin for 24 h. Cells were stained with Hoechst 33342 and then examined under a light microscope. Click here for file

Additional file 3 **Table S1 Q value**. Q values are shown. Q = 0.85-1.15 indicates simple addition; Q > 1.15 indicates synergism. Click here for file
