# Supplementary figures and images for: Curcumin reduces expression of Bcl-2, leading to apoptosis in daunorubicin-insensitive CD34+ acute myeloid leukemia cell lines and primary sorted CD34+ acute myeloid leukemia cells
Source: J Transl Med. 2011 May 19;9:71. doi: 10.1186/1479-5876-9-71 (PMC3118333; doi:10.1186/1479-5876-9-71)

A

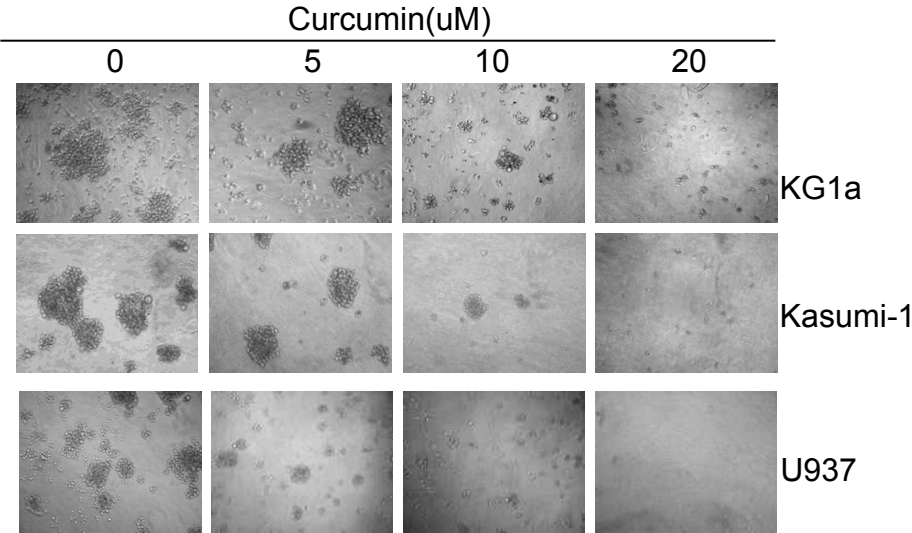

B

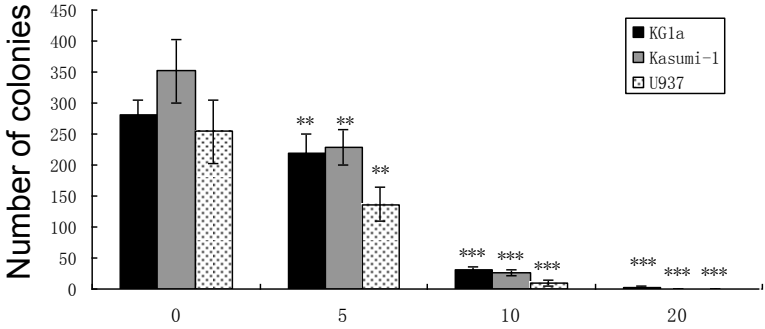

Supplement: Additional file 1 — Figure S1 Curcumin inhibited clonogenic growth. (A) The colonies (containing ≥50) were counted after 14 days by light microscopy (magnification ×40). (B) Results show numbers of colonies in the curcumin-treated group expressed as a percentage of number of colonies in the DMSO-treated group. The graph displays the means ± SD of three independent experiments. [file 1479-5876-9-71-S1.PDF]

A

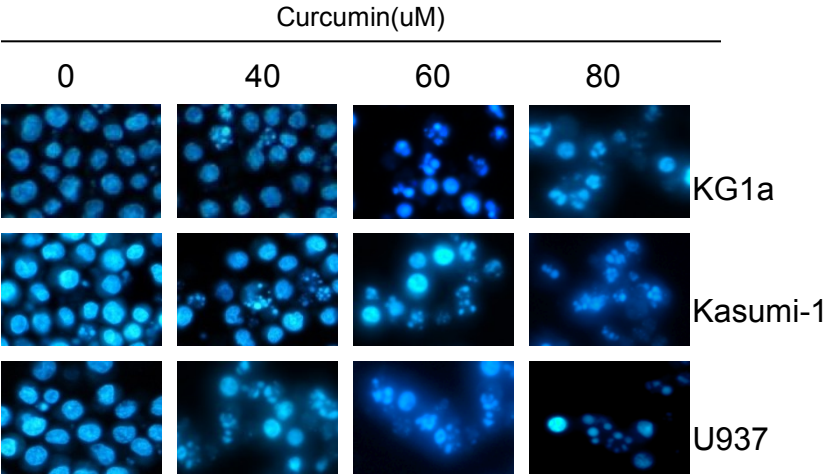

Supplement: Additional file 2 — Figure S2 Morphological changes in nuclei in curcumin-treated cells. (A) KG1a, Kasumi-1 and U937 cells were incubated with the indicated concentrations of 0, 40, 60, and 80 μM curcumin for 24 h. Cells were stained with Hoechst 33342 and then examined under a light microscope. [file 1479-5876-9-71-S2.PDF]
