# Supplementary material for: Curcumin reduces expression of Bcl-2, leading to apoptosis in daunorubicin-insensitive CD34+ acute myeloid leukemia cell lines and primary sorted CD34+ acute myeloid leukemia cells
Source: J Transl Med. 2011 May 19;9:71. doi: 10.1186/1479-5876-9-71 (PMC3118333; doi:10.1186/1479-5876-9-71)
Supplement: Additional file 3 — Table S1 Q value. Q values are shown. Q = 0.85-1.15 indicates simple addition; Q > 1.15 indicates synergism. [file 1479-5876-9-71-S3.PDF]

KG1a cells

Curcumin (μM)	DNR (μg/ml)	Q value	Result
20	0.1	0.99	addition
40	0.2	1.49	synergism
80	0.4	1.39	synergism

Kasumi-1 cells

Curcumin (μM)	DNR (μg/ml)	Q value	Result
20	0.1	1.77	synergism
40	0.2	1.39	synergism
80	0.4	1.36	synergism
